# Corticospinal excitability to the biceps brachii and its relationship to postactivation potentiation of the elbow flexors

**DOI:** 10.14814/phy2.13265

**Published:** 2017-04-28

**Authors:** Brandon W. Collins, Laura H. Gale, Natasha C. M. Buckle, Duane C. Button

**Affiliations:** ^1^ Human Neurophysiology LaboratorySchool of Human Kinetics and RecreationNewfoundland and LabradorCanada; ^2^ BioMedical SciencesFaculty of MedicineMemorial UniversitySt. John'sNewfoundland and LabradorCanada

**Keywords:** Motoneurone, postexercise depression, postexercise facilitation, transcranial magnetic stimulation, transmastoid electrical stimulation

## Abstract

We examined the effects of a submaximal voluntary elbow flexor contraction protocol on measures of corticospinal excitability and postactivation potentiation of evoked muscle forces and if these measures were state‐dependent (rest vs. voluntary muscle contraction). Participants completed four experimental sessions where they rested or performed a 5% maximum voluntary contraction (MVC) of the elbow flexors prior to, immediately, and 5 min following a submaximal contraction protocol. During rest or 5% MVC, transcranial magnetic stimulation, transmastoid electrical stimulation, electrical stimulation of biceps brachii motor point and Erb's point were elicited to induce motor‐evoked potentials (MEPs), cervicomedullary MEPs (CMEPs), potentiated twitch (PT) force, and maximal muscle compound action potential (*M*_max_), respectively prior to, immediately, and 5 min postcontraction protocol. MEP amplitudes increased (215 and 165%M_max_, *P *≤* *0.03) only at 1 and 6s postcontraction protocol, respectively during rest but not 5% MVC. CMEP amplitudes decreased during rest and 5% MVC (range:21–58%*M*_max_, *P *≤* *0.04) for up to 81 sec postcontraction protocol. Peak twitch force increased immediately postcontraction protocol and remained elevated for 90 sec (range:122–147% increase, *P *<* *0.05). There was a significant positive correlation between MEP and PT force during rest (*r *=* *0.88, *P *=* *0.01) and a negative correlation between CMEP and PT force during rest (*r *=* *−0.85, *P *<* *0.02 and 5% MVC (*r *=* *−0.96, *P *<* *0.01) immediately postcontraction protocol. In conclusion, the change in corticospinal and spinal excitability was state‐ and time‐dependent whereas spinal excitability and postactivation potentiation were time‐dependent following the contraction protocol. Changes in corticospinal excitability and postactivation potentiation correlated and were also state‐dependent.

## Introduction

The malleability of central nervous system excitability and evoked muscle twitch force is dependent on muscle contractile history. For example, immediately following and well beyond the cessation of a voluntary contraction(s) of a given muscle there is altered excitability of the corticospinal tract projecting to that muscle (Gandevia et al. 1999; Norgaard et al. 2000; Balbi et al. 2002; Aboodarda et al. 2015) and the evoked twitch force of that given muscle (Vandervoort et al. 1983; Kufel et al. 2002; Behm et al. 2004).

Postexercise facilitation and postexercise depression (i.e., an increase and decrease, respectively in the evoked potential amplitude) of corticospinal excitability occurs in fresh and fatigued muscles including the biceps brachii (Sacco et al. 1997; Gandevia et al. 1999; Norgaard et al. 2000; Humphry et al. 2004; Aboodarda et al. 2015), first dorsal interosseous (McDonnell and Ridding 2006; Giesebrecht et al. 2011; Teo et al. 2012), extensor carpi radialis (Samii et al. 1996), flexor carpi radialis (Brasil‐Neto et al. 1993), thenar (Zanette et al. 1995; Balbi et al. 2002) and soleus (Norgaard et al. 2000) following contraction protocols of varying degrees of contraction intensities and durations. Motor evoked potential (MEP) amplitudes can double in size (Samii et al. 1996; Norgaard et al. 2000; Balbi et al. 2002; Aboodarda et al. 2015), whereas cervicomedullary MEP (CMEP) amplitudes can be reduced to half its size following submaximal muscle contractions compared with precontraction values (Aboodarda et al. 2015). The aforementioned studies found changes in corticospinal excitability of a resting muscle following a contraction protocol. Corticospinal excitability, however, differs depending on the state (i.e., during rest or a voluntary muscle contraction) in which it is measured. MEP and CMEP amplitudes increase during muscle contraction (Hess et al. 1987; Darling et al. 2006; Martin et al. 2009; Pearcey et al. 2014; Philpott et al. 2015) and CMEP amplitudes are less depressed following fatigue when they are measured during a 5% MVC compared to rest (Petersen et al. 2003). Thus, it is likely that postexercise facilitation and postexercise depression following voluntary contractions differ when measured during rest compared to a voluntary muscle contraction.

Enhanced evoked twitch force also occurs following voluntary muscle contractions. This postactivation potentiation is due to an enhancement in the contractile mechanical performance of the muscle following the contractions (MacIntosh 2010). Postactivation potentiation occurs following voluntary contractions at intensities as low as 20–50% MVC as demonstrated in the elbow extensors (Smith et al. 2011), plantar flexors (Fukutani et al. 2014), thumb abductors (Fukutani et al. 2014) and knee extensors (Dolmage and Cafarelli 1991; Place et al. 2005; Morana and Perrey 2009).

Corticospinal excitability and postactivation potentiation are important mechanisms that underlie the development of muscle force. However, no previous study has attempted to determine whether there is a relationship between these measures of neuromuscular excitability. Motor unit recordings, which measure motoneurone output (i.e., firing frequency) to the muscle during voluntary contraction have shown that motor unit discharge rates were decreased in the presence of postactivation potentiation in the triceps brachii (Klein et al. 2001) and the tibialis anterior (Inglis et al. 2011). Postactivation potentiation of the muscle may allow for a reduction in motor unit discharge rates. Currently, it is not known if a relationship between corticospinal excitability and postactivation potentiation exists.

The purpose of this study was (1) to assess the effects of repeated, submaximal voluntary contractions of the elbow flexors on corticospinal excitability to the biceps brachii and postactivation potentiation of the elbow flexors during rest and a slight contraction (5% MVC) and (2) determine if a state‐dependent relationship existed between corticospinal excitability and postactivation potentiation. We hypothesized that (1) there would be postexercise facilitation of corticospinal excitability (i.e., MEPs), postexercise depression of spinal excitability (i.e., CMEPs) and postactivation potentiation of the elbow flexors postcontraction protocol and (2) there would be a negative relationship between spinal excitability and postactivation potentiation during 5% MVC.

## Materials and Methods

### Participants

University aged resistance‐trained males (178.65 ± 7.43 cm, 82.47 ± 12.38 kg, 24.11 ± 5.25 years) were recruited for the study (*n* = 9 for *experiment A* and *n* = 6 for *experiment B*). We chose to recruit only resistance‐trained males because corticospinal excitability is training‐dependent (Carroll et al. 2002; Falvo et al. 2010; Pearcey et al. 2014; Philpott et al. 2015). Participants were verbally informed of the procedures to be used during testing, and all gave informed written consent and completed a magnetic stimulation safety checklist to screen for potential contraindications with magnetic stimulation procedure (Rossi et al. 2011). The study was approved by the Memorial University of Newfoundland Interdisciplinary Committee on Ethics in Human Research (#20161806‐HK) and was in accordance with the Tri‐Council guidelines in Canada with full disclosure of potential risks to participants.

### Elbow flexor force

**Figure 1:**
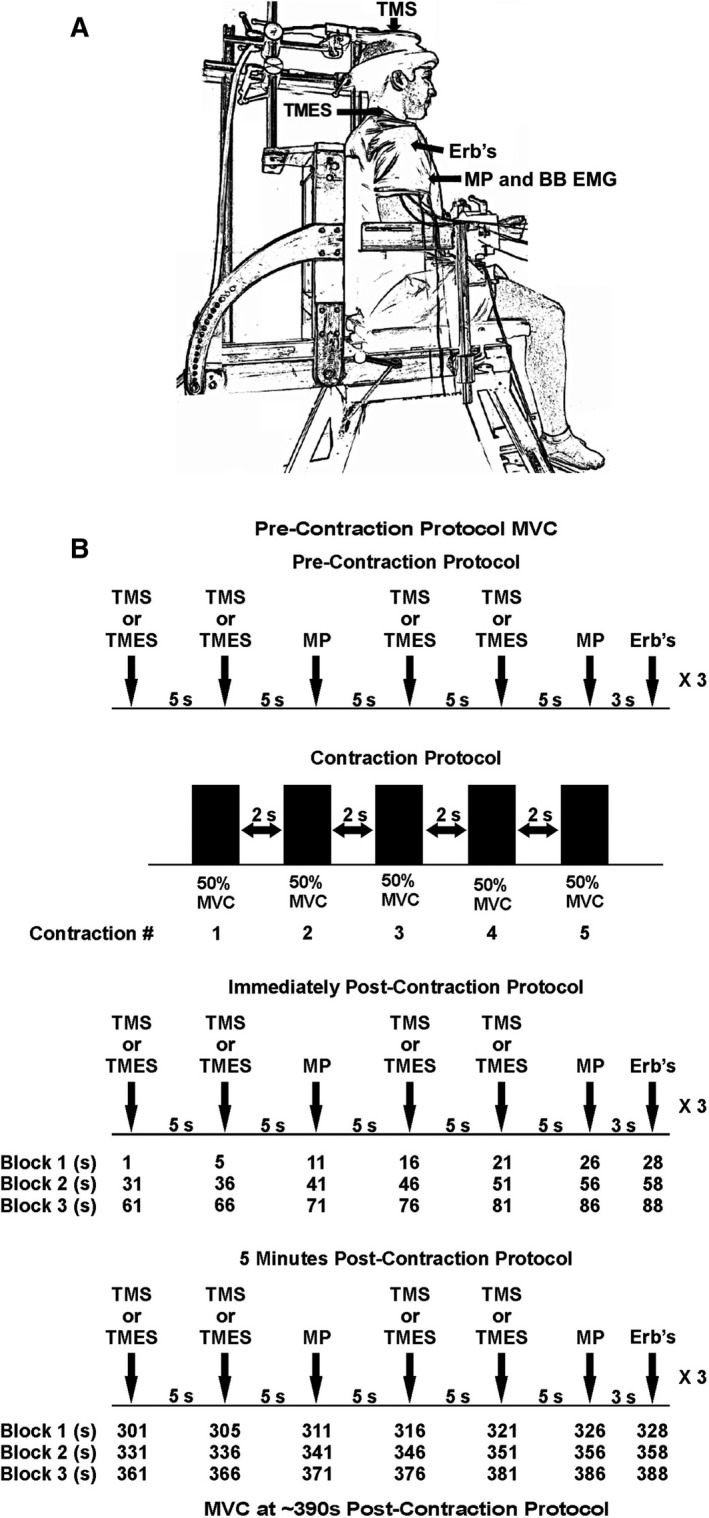
Experimental set‐up and general procedure. (A) During rest or elbow flexion at 5% MVC, TMS was applied over vertex to activate the motor cortex of the contralateral hemisphere. TMES was applied between the mastoid processes, nerve stimulation at Erb's point and muscle stimulation at biceps brachii motor point. Evoked potentials were recorded from the biceps brachii and peak twitch force was recorded from the elbow flexors. (B) The experimental protocol consisted of TMS (*experiment A*), TMES (*experiment B*) and motor point stimulation at 5 sec intervals and Erb's point stimulation at 28 sec during a 30 sec time block. The block was then repeated 3 times; pre, immediately and 5‐min postcontraction protocol. The contraction protocol itself consisted of 5, 2 sec contractions at 50% MVC with 2 sec rest between each contraction. TMS, Transcranial magnetic stimulation; TMES, Transmastoid electrical stimulation; MVC, maximum voluntary contraction.

Participants were seated in an upright position, with hips and knees flexed at 90 ˚, and head and chest strapped in place to minimize movement (see Fig. 1A). Both arms were slightly abducted with elbows resting on padded support at an angle of 90 ˚. The forearms were held horizontal in a position midway between neutral and supination, and placed in a custom‐made orthosis that was connected to a load cell (Omegadyne Inc., Sunbury, OH). The load cell detected force output from the dominant elbow flexors, which was amplified (×1000) (CED 1902, Cambridge Electronic Design Ltd., Cambridge, UK) and displayed on a computer screen. Data was sampled at 2000 Hz. Visual feedback was given to all participants during contractions.

### Electromyography

Electromyography (EMG) activity was recorded from the dominant biceps brachii muscle using surface EMG recording electrodes (MediTrace Ag‐AgCl pellet electrodes, disc shaped and 10 mm in diameter, Graphic Controls Ltd., Buffalo, NY). Electrodes were placed 2 cm apart over the midpoint of the muscle belly. A ground electrode was placed over the lateral epicondyle of the dominant knee. Skin preparation for all recording electrodes included shaving to remove excess hair and cleaning with an isopropyl alcohol swab to remove dry epithelial cells. EMG signals were amplified (×1000) (CED 1902) and filtered using a 3‐pole Butterworth filter with cut‐off frequencies of 10–1000 Hz. All signals were analog‐digitally converted at a sampling rate of 5 kHz using a CED 1401 interface.

### Stimulation conditions

#### Transcranial magnetic stimulation

Transcranial magnetic stimulation (TMS)‐evoked MEPs were used to measure corticospinal excitability from the relaxed dominant biceps brachii muscle as well as during a 5% contraction, using a transcranial magnetic stimulator (Magstim 200, maximum output 2.0 Tesla) with a circular coil (13 cm outside diameter) directly placed over the vertex (Forman et al. 2014; Pearcey et al. 2014, 2016; Aboodarda et al. 2015; Philpott et al. 2015). The coil was placed horizontally over the vertex with the direction of the current flow to specifically activate the left or right cortex depending on arm dominance. Electrical currents flowed in an anticlockwise direction through the circular coil. Vertex was located by marking the measured halfway points between the nasion and inion and tragus to tragus. The intersection of these halfway points was defined as the vertex. The coil was placed horizontally over the vertex so that the direction of the current flow in the coil preferentially activated the motor cortex for the activation of biceps brachii. Stimulation intensity (35–75% of maximal stimulator output) was adjusted to elicit a threshold response of ≥50 *μ*V, in 50% of the trials (i.e., four out of eight trials) in the biceps brachii during rest and an identifiable response during 5% MVC. The mean stimulator output was then increased by 20% above that used to determine threshold for the remainder of the experiment (Forman et al. 2014; Aboodarda et al. 2015).

#### Transmastoid electrical stimulation

Stimulation was applied via surface electrodes placed over the mastoid processes and current was passed between them (100 *μ*sec duration, 100–350 mA; model DS7AH, Digitimer Ltd, Welwyn Garden City, UK). The latencies of all CMEPs were monitored because evoked stimulation to the mastoid processes can activate axons near the ventral roots which subsequently decreases the onset latency of the CMEP by ~2 msec (Taylor 2006). Based on the latencies (~7.8 ± 0.8 msec), none of the CMEPs were contaminated by the activation of ventral roots. Stimulation intensity was adjusted to elicit a response in 50% of the trials (i.e., four out of eight trials) in the dominant biceps brachii either during rest or during 5% MVC. Stimulator intensity was then increased by 20% above that used to determine threshold for the remainder of the experiment.

#### Brachial plexus stimulation

Stimulation of the brachial plexus (i.e., Erb's point) was used to measure maximal compound muscle action potential (*M*_max_). Erb's point was electrically stimulated via a cathode on the skin in the supraclavicular fossa and an anode on the acromion process. Current pulses were delivered as a singlet (200 *μ*sec duration, 150–350 mA). The electrical current was gradually increased until *M*_max_ of the dominant biceps brachii was observed. Stimulator intensity was then increased by 20% above that used to determine *M*_max_ for the remainder of the experiment (Aboodarda et al. 2015).

#### Motor point stimulation

Biceps brachii motor point stimulation was used to assess evoked contractile properties. The motor point electrode was placed just proximal and medial to the midpoint of the muscle belly. Electrical stimulation was delivered via a cathode placed on the skin over the biceps motor point and an anode on the brachii distal tendon. Current pulses were delivered as a doublet (10 msec apart, 100 *μ*sec duration, 175–300 mA). The electrical current was gradually increased until there was no longer an increase in the twitch force of the dominant elbow flexors. Stimulator intensity was then increased by 10% above that used to determine maximal twitch force for the remainder of the experiment (Allen et al. 1998).

### Experimental set‐up A

Participants completed a familiarization session and two randomized experimental sessions with 24–48 h between sessions.

#### Contraction protocol

The contraction protocol consisted of five submaximal and intermittent contractions of the elbow flexors performed at 50% MVC, with 2 sec of contraction followed by 2 sec of rest. The 50% MVC target force was displayed on a computer screen and participants were asked to match their force output to the target force.

#### Familiarization session

Participants completed MVCs and the contraction protocol. Participants also received the different forms of stimulation used (i.e., TMS, Transmastoid electrical stimulation (TMES), Erb's point and motor point).

#### Experimental session 1

Participants performed a MVC of the elbow flexors prior to the precontraction protocol. Approximately 15‐ min of rest was provided following the initial MVC and the stimulation intensities for MEP, *M*_max_ and PT force were determined. The experimental procedures then began. Pre and postcontraction protocol measurements consisted of three sets of 30 sec duration trials, with each 30 sec‐trial consisting of a twice repeated sequence of stimulations in the same order (TMS, TMS, and motor point). Each stimulus was separated by 5s. Erb's Point stimulation occurred at 28 sec within each 30 sec trial. Each series of neuromuscular testing lasted 90 sec and included 12 transcranial, 6 motor point and 3 Erb's point stimulations. This sequence was performed pre, immediately, and 5‐ min postcontraction protocol. Approximately 6.5‐min following the contraction protocol another elbow flexor MVC was performed to assess maximum force production capability.

#### Experimental session 2

Participants performed the exact same protocol as experimental session 1, however, participants continuously held a contraction with no rest at 5% of their MVC during each 90 sec block (pre, immediately and 5‐min postcontraction protocol) of testing, as well as during determination of stimulation intensities.

### Experimental set‐up B

Six participants from *experiment A* completed two experimental sessions in *experiment B*. Experimental sessions 1 and 2 in *experiment B* were identical to those outlined in *experiment A*, with one major difference. In *experiment B*, TMS was replaced with TMES to assess spinal excitability. All other portions of the protocol remained the same. See Figure 1 A and B for experimental set‐up and protocol details.

### Data analysis

**Figure 2:**
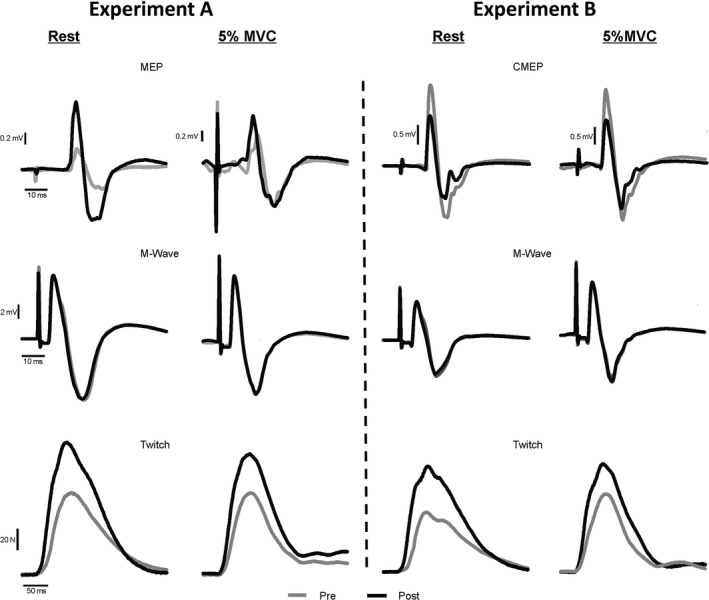
Raw data illustrating changes in corticospinal, spinal and muscle compound action potential (*M*_max_) responses of the biceps brachii and twitch forces of the elbow flexors during rest and 5% MVC following the contraction protocol. Individual raw data traces from a single subject for *experiment A* (left panel) *and experiment B* (right panel) for MEP, cervicomedullary evoked potentials, *M*_max_ and PT force. The gray line represents the last response for each MEP, CMEP,* M*_max_ and PT force recorded during the precontraction protocol and the black line represents the first response for each MEP, CMEP,* M*_max_ and PT force recorded immediately postcontraction protocol. PT, peak twitch.

The peak‐to‐peak amplitudes were measured for MEP, CMEP, and *M*_max_ responses throughout each testing block. All MEPs and CMEPs were normalized to the recorded *M*_max_ within the same 30 sec trial and all MEP and CMEP data reported in the results section are expressed as a percentage of *M*_max_. Peak twitch (PT) force of the elbow flexors was defined as the peak amplitude of the twitch force. Figure 2 shows raw data of one participant during rest and 5% MVC which includes the last (gray line) MEP (left top panel) or CMEP (right top panel), *M*_max_ (middle panel) and PT force (bottom panel) recorded precontraction protocol and the first (black line) MEP or CMEP, *M*_max_ and PT force recorded immediately postcontraction protocol. To determine if central drive to the biceps brachii was similar within each experimental session, mean biceps brachii root mean square (RMS) EMG amplitude was measured for 100 msec prior to each stimulus. RMS EMG was also calculated over a 1 sec epoch during each 50% MVC within each experimental session. Because there was no difference between MEP and CMEP amplitudes and PT forces precontraction protocol, they were averaged and each MEP, CMEP, and PT response recorded postcontraction protocol was compared with the average as previously done in our lab (Aboodarda et al. 2015).

For correlations, 2 MEP and 2 CMEP amplitudes were averaged from every 15 sec block during the 90 sec of recording immediately postcontraction protocol and correlated with the following PT force that was recorded in that given 15 sec. MEP and CMEP correlations to PT force were based on MEP, CMEP and PT force amplitude percentage change (at the time points immediately postcontraction protocol) from the precontraction protocol average.

### Statistical analysis

**Figure 3:**
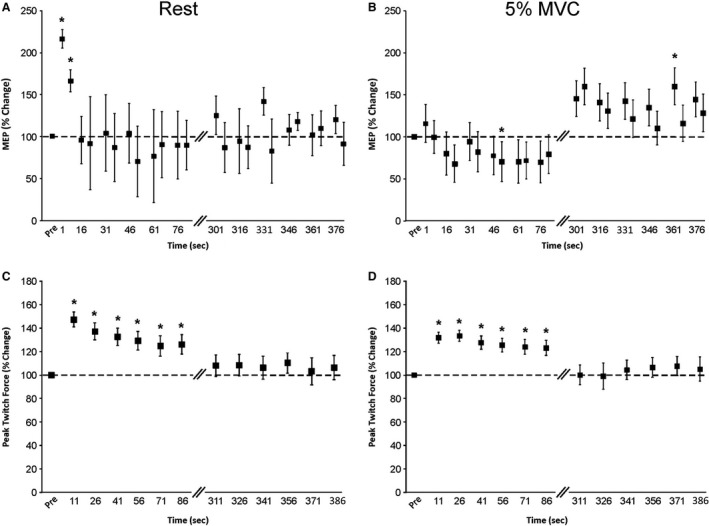
Changes in corticospinal excitability and peak twitch force following 5, 50% MVCs of the elbow flexors (*experiment A*). Percentage change from the precontraction average for MEPs during (A) rest and (B) 5% MVC of the biceps brachii and peak twitch forces during (C) rest and (D) 5% MVC of the elbow flexors. The horizontal dashed line in each panel represents the average of all precontraction values. Pairs of diagonal lines on the *x*‐axis represent the 3.5‐min time period between data recording. Each data point represents the group percentage change mean ± SE for all time points immediately and 5‐min postcontractions. Asterisk (*) indicates a significant difference (*P* < 0.05) from precontraction protocol in (A) and (B) and all other time points in (C) and (D). MVC,maximum voluntary contraction; MEP, motor evoked potentials; PT, peak twitch.

**Figure 4:**
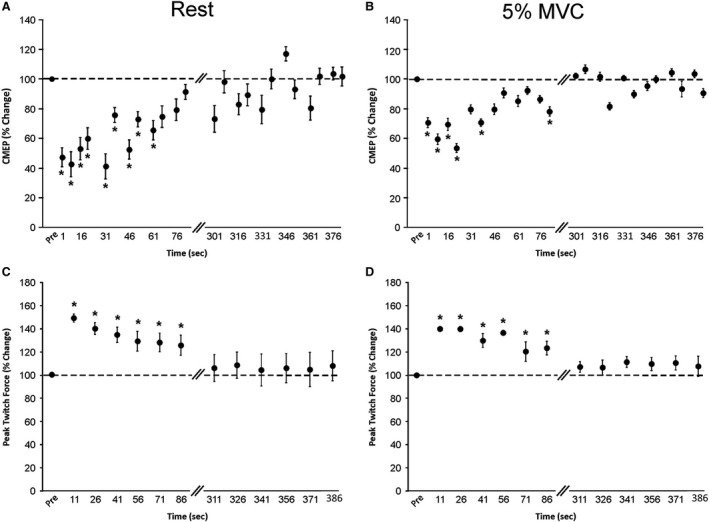
Changes in spinal excitability and peak twitch force following 5, 50% MVCs of the elbow flexors (*experiment B*). Percentage change from precontractions for cervicomedullary motor evoked potentials (CMEPs) during (A) rest and (B) 5% MVC of the biceps brachii and peak twitch forces during (C) rest and (D) 5% MVC of the elbow flexors. The horizontal dashed line in each panel represents the average of all precontraction values. Pairs of diagonal lines on the *x*‐axis represent the 3.5‐min time period between data recording. Each data point represents the group percentage change mean ± SE for all time points immediately and 5‐min postcontractions. Asterisk (*) indicates a significant difference (*P* < 0.05) from precontraction protocol in (A) and (B) and all other time points in (C) and (D). MVC,maximum voluntary contraction.

**Figure 5:**
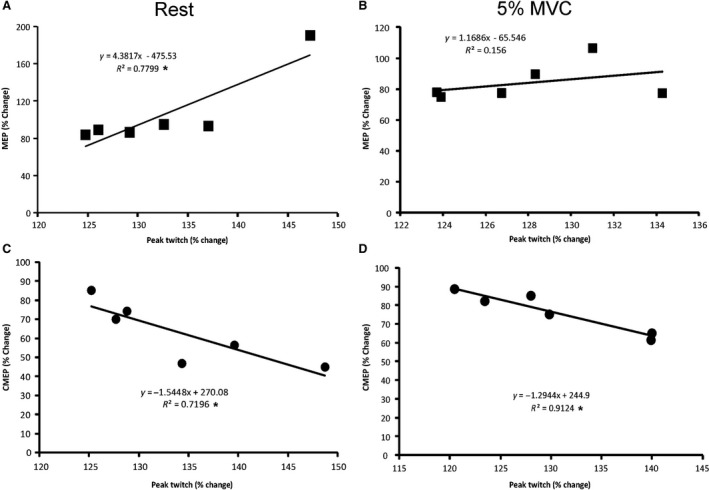
Correlations between central excitability and peak twitch forces following 5, 50% MVCs of the elbow flexors. Relationship between MEPs and peak twitch forces during (A) rest and (B) 5% MVC of the elbow flexors (*experiment A*). Relationship between CMEPs and peak twitch forces during (C) rest and (D) 5% MVC of the elbow flexors (*experiment B*). Each data point represents the group average for each *experiment A* (*n* = 9) and *B* (*n* = 6). The slopes and *R* ^2^ values are illustrated for each group. Asterisk (*) indicates a significant correlation (*P* < 0.05). MVC,maximum voluntary contraction; MEP, motor evoked potentials; CMEPs, cervicomedullary motor evoked potentials; PT, peak twitch.

Statistical analyses were computed using SPSS software (SPSS 22.0, IBM Corporation, Armonk, NY). Assumptions of sphericity (Mauchley test) and normality (Shapiro‐Wilk test) were tested for all dependent variables. If the assumption of sphericity was violated, the corrected value for non‐sphericity with Greenhouse‐Geisser epsilon was reported. All data were normally distributed. We did not compare corticospinal excitability responses recorded during rest and 5% MVC because corticospinal excitability is different between resting and muscle contraction conditions, nor did we compare MEPs to CMEPs because their amplitudes were not matched (see Methodological Considerations in Discussion), thus a one‐way ANOVA with repeated measures was performed on all dependent variables to examine within group differences. Statistical significance was set at *P *<* *0.05 and a Bonferroni post hoc test was performed to test for significant differences between time points. Pearson product‐moment correlation coefficients between percent changes in corticospinal excitability and PT force were also performed. Absolute values are reported in text and mean percentage change from the averaged precontraction protocol value in Figures (3, 4 and 5). All data are reported as means ± SE.

## Results

There was no significant main effect of time on precontraction protocol MEP (*experiment A*,* P *=* *0.56 and *P *=* *0.30), CMEP (*experiment B*,* P *=* *0.17 and *P *=* *0.56) or PT force (*experiment A, P *=* *0.29 and *P *=* *0.14 and *experiment B, P *=* *0.35 and *P *=* *0.14) amplitudes during rest and 5% MVC, respectively. There was no significant main effect of time on prestimulus EMG, thus overall central drive was similar within each experimental session during rest (*experiment A*,* P *=* *0.74 and *experiment B*,* P *=* *0.59) and 5% MVC (*experiment A*,* P *=* *0.38 and *experiment B*,* P *=* *0.24).

### Corticospinal excitability

#### Rest

There was a significant main effect for time (*P *<* *0.01) on individual MEP amplitudes (Fig. 3A). MEP amplitudes were significantly higher at 1s (11.1 ± 6.8% *M*_max_; *P *<* *0.05) and 6 sec (7.5 ± 3.3% *M*_max_; *P *<* *0.01) postcontraction protocol compared to the averaged precontraction protocol MEP amplitude (4.7 ± 1.2% *M*_max_).

#### *5% MVC*

There was a significant main effect for time (*P *<* *0.01) on individual MEP amplitudes (Fig. 3B). MEP amplitude was significantly (*P *<* *0.05) lower at 51s (15.3 ± 4.0% *M*_max_) and significantly (*P *<* *0.05) higher at 361 sec (29.2 ± 8.7% *M*_max_) postcontraction protocol compared to the averaged precontraction protocol MEP amplitude (16.9 ± 3.1% *M*_max_).

### Spinal excitability

#### Rest

There was a significant main effect for time (*P *<* *0.01) on individual CMEP amplitudes (Fig. 4A). The CMEP amplitudes at 1, 5, 16, 21, 31, 36, 46, 51, 61 sec (ranging from 10.1 ± 3.0–15.3 ± 3.8% *M*_max_) postcontraction protocol were significantly (*P *<* *0.05) lower than the averaged precontraction protocol CMEP amplitude (22.5 ± 6.2% *M*_max_).

#### *5%* MVC

There was a significant main effect for time (*P *<* *0.01) on individual CMEP amplitudes (Fig. 4B). The CMEP amplitudes at 1, 5, 21, 36, 76 and 81s (ranging from 10.3 ± 1.6–17.6 ± 3.1% *M*_max_) postcontraction protocol were significantly (*P *<* *0.03) lower than the averaged precontraction protocol CMEP amplitude (20.6 ± 2.5% *M*_max_).

### Peak twitch force and *M*_max_

#### Rest

There was a significant main effect (*experiment A*,* P *<* *0.01 and *experiment B*,* P *<* *0.01) of time on individual PT forces (Figs. 3C and 4C, respectively). In *experiment A*, the PT forces at 11, 26, 41, 56, 71, and 86 (range; 80.9 ± 8.4–91.8 ± 8.6 N) postcontraction protocol were significantly (*P *<* *0.04) higher than all other PT forces (range: 64.5 ± 6.0–77.1 ± 7.0 N) (Fig. 3C). In *experiment B*, the PT forces at 11, 26, 41, 56, 71, and 86 sec (range: 86.1 ± 7.7–99.8 ± 4.6 N) were significantly (*P *<* *0.05) higher than all other PT forces (range: 69.6 ± 6.7–74.7 ± 5.4 N) (Fig. 4C).

#### *5%* MVC

There was a significant main effect (*experiments A* and *B*,* P *<* *0.01) of time on individual PT forces (Figs. 3D and 4D, respectively). In *experiment A* the PT forces at 11, 26, 41, 56, 71 and 86 sec (range: 86.3 ± 6.4–93.5 ± 5.5 N) postcontraction protocol were significantly (*P *<* *0.01) higher than all other PT forces (range: 71.0 ± 5.7–76.1 ± 6.5 N) (Fig. 3D). In *experiment B* the PT forces at 11, 26, 41, 56, 71 and 86 sec (range: 87.4 ± 6.4–97.6 ± 2.2 N) were significantly (*P *<* *0.05) higher than all other PT forces (range: 70.9 ± 4.4–78.4 ± 4.2 N) (Fig. 4D).

There was no significant main effect (*experiment A, P *=* *0.86 and *P *=* *0.67 and *experiment B*,* P *=* *0.113 and *P *=* *0.297) for the contraction protocol on *M*_max_ amplitudes (*experiment A* range;12.9 ± 5.4–13.5 ± 6.2 and 8.4 ± 4.2–9.4 ± 5.7 mV and *experiment B* range: 8.9 ± 5.9–10.9 ± 6.8 and 9.2 ± 6.9–9.9 ± 6.2 mV) during rest and 5% MVC, respectively.

### Correlations between corticospinal excitability (MEPs and CMEPs) and peak twitch force measurements

In *experiment A*, there was a significant positive correlation between MEP and PT force immediately postcontraction protocol during rest (*r *=* *0.88, *P *=* *0.01) (Fig. 5A) but not 5% MVC (*r *=* *0.40, *P *=* *0.22) (Fig. 5B). In experiment B, there was a significant negative correlation between CMEPs and PT forces immediately postcontraction protocol during rest (*r *=* *−0.85, *P *<* *0.02) (Fig. 5C) and 5% MVC (*r *=* *−0.96, *P *<* *0.01) (Fig. 5D). No correlations occurred 5‐min postcontraction protocol.

### MVC and EMG

There was no significant main effect (*experiment A*,* P *=* *0.13 and *P *=* *0.25 and *experiment A, P *=* *0.36 and *P *=* *0.44) for the contraction protocol on MVC force pre and postcontraction protocol (*experiment A*, 470.1 ± 33.3 and 461.4 ± 25.5 N and *experiment B*, 466.8 ± 82.1 and 453.4 ± 70.9 N) or 5% MVC (*experiment A*, 473.3 ± 28.1 and 449.7 ± 30.1 N and *experiment B*, 446.1 ± 72.1 and 442.9 ± 71.8 N), respectively.

There was no significant main effect for (*experiment A*,* P *=* *0.62 and *P *=* *0.49 and *experiment B, P *=* *0.96 and *P *=* *0.92) for contraction number during the contraction protocol on EMG between 50% MVC 1 and 5 during rest (*experiment A*, 0.57 ± 0.13 and 0.67 ± 0.14 mV and *experiment B*, 0.59 ± 0.16 and 0.58 ± 0.13 mV) or 5% MVC (*experiment A*, 0.52 ± 0.09 and 0.62 ± 0.11 mV and *experiment B*, 0.62 ± 0.15 and 0.64 ± 0.16 mV), respectively.

## Discussion

We demonstrated that the interaction between postexercise facilitation, postexercise depression and postactivation potentiation following brief, nonfatiguing, submaximal and intermittent 50% MVC contractions of the elbow flexors result in a state‐dependent; (1) change in corticospinal excitability to the biceps brachii but not spinal excitability or postactivation potentiation of the elbow flexors and (2) relationship between corticospinal excitability and postactivation potentiation but not spinal excitability and postactivation potentiation. There was no change in MVC force output from pre to postcontraction protocol or EMG during the contraction protocol. Also, prestimulus EMG was similar throughout each experimental session. Thus, the changes in postexercise facilitation, postexercise depression and postactivation potentiation and their relationship were not due to fatigue or changes in central drive to the muscle following the contraction protocol.

### Corticospinal excitability

During rest, the MEP amplitude facilitation (215 and 165%) was transient as they returned to baseline within 8–16 sec postcontraction protocol, which is a similar percentage change and time frame as previously reported (Norgaard et al. 2000; Aboodarda et al. 2015). Previous work has shown a similar postexercise facilitation of MEP amplitudes following different contraction durations (2–30 sec) and intensities (10–100% MVC) of different musculature, including the thenar muscles, extensor carpi radialis, soleus and biceps brachii (Samii et al. 1996; Norgaard et al. 2000; Balbi et al. 2002; Aboodarda et al. 2015). The main mechanism of postexercise facilitation is probably potentiation (posttetanic, short‐term and or long‐term) whereby brief contractions may facilitate synaptic transmission and neurotransmitter release within motor cortex circuitry for a period of time (Samii et al. 1996). Postexercise depression of MEP amplitudes has been shown following rapid contractions and sensory motor tasks (i.e., peg board test) of the first dorsal interosseous (McDonnell and Ridding 2006; Teo et al. 2012), abductor pollicis brevis (Teo et al. 2012) and right thenar eminence (Zanette et al. 1995). Mechanisms for postexercise depression may include reduced neurotransmitter levels, reduced excitability of intracortical networks, enhanced excitability of inhibitory intracortical networks and long‐term depression (Zanette et al. 1995; Samii et al. 1996; Teo et al. 2012).

Postexercise facilitation of MEPs did not occur during 5% MVC. During the voluntary drive to produce 5% MVC, potentiation may no longer be evident because the supraspinal drive may overcome the potentiating effects. Studies have shown that MEP amplitude depression was greater when recorded during active (i.e., muscle contraction) than passive (i.e., no muscle contraction) movements (Miyaguchi et al. 2013) because during a muscle contraction the activation of Ia afferents and cutaneous mechanoreceptors (Coxon et al. 2005) may act to suppress the MEP amplitude. The lack of postexercise facilitation of MEP amplitude in the current study during the slight contraction could be, in part, explained by the activation of these receptors that is not present in a quiescent muscle or voluntary drive itself may mask the intracortical potentiation. Based on the aforementioned literature and the findings here, whether MEPs are facilitated or depressed following contractions appears to be task‐ and state‐dependent.

### Supraspinal excitability

There was a transient increase in MEP and decrease in CMEP amplitudes during rest but no change in MEP and a decrease in CMEP amplitudes during 5% MVC. It could be concluded that postexercise facilitation followed by a postexercise depression of supraspinal excitability occurred at rest whereas during 5% MVC either postexercise depression of supraspinal excitability occurred or there was an increase in supraspinal excitability but it was masked by the decrease in spinal excitability (Pearcey et al. 2014). However, this is speculative because we did not match MEP to CMEP in the current study. A recent study (Aboodarda et al. 2015) that used a similar contraction protocol did match MEP and CMEP amplitudes and made a ratio of MEP/CMEP to illustrate changes in supraspinal excitability. They found a transient postexercise facilitation followed by a postexercise depression of supraspinal excitability (Aboodarda et al. 2015), which supports our findings. Increased MEPs and decreased H‐reflex amplitudes occur in the soleus following submaximal plantar flexor contractions illustrating postexercise facilitation of supraspinal excitability (Norgaard et al. 2000) and similar results have been reported by others (Samii et al. 1996; Balbi et al. 2002). The increased supraspinal excitability following the contraction protocol may be due to decreased short‐interval cortical inhibition (SICI)(Garry et al. 2004) or increased potentiation as previously discussed. SICI and intracortical facilitation (ICF) is reduced during a nonfatiguing contraction compared to rest (Ridding et al. 1995) which may explain the decreased supraspinal excitability during 5% MVC in the current study. SICI and ICF are also reduced during submaximal contractions of the elbow flexors (Hunter et al. 2016).

### Spinal excitability

There was a postexercise depression of CMEP amplitudes (21–58%) that lasted up to 81 sec postcontraction protocol, which is similar to previously reported findings (Aboodarda et al. 2015). Postexercise depression of spinal excitability following contractions may be due to depletion of readily available neurotransmitter stores at the synapses along the corticospinal pathway (Petersen et al. 2003) or activity‐dependent changes at the spinal motoneurone (Button et al. 2006; Heckman et al. 2008; Khan et al. 2012; MacDonell et al. 2012). A reduction in soleus H‐reflex also occurs following 50% MVC of the plantar flexors indicating that postexercise depression of spinal excitability may also be due to increased presynaptic inhibition (Norgaard et al. 2000). Furthermore, the intensity of a conditioning contraction and the firing frequencies of descending drive from the cortex to the motoneurone may also contribute to synaptic fatigue at the cortico‐motoneuronal junction (Petersen et al. 2003), subsequently leading to postexercise depression of spinal excitability. Although the contraction protocol utilized in this study was not intense enough to induce neuromuscular fatigue, it may have led to some degree of synaptic fatigue (i.e., reduced neurotransmitter release). On the other hand, studies have not shown postexercise depression of spinal excitability of the flexor carpi radialis and thenar muscles at rest following submaximal wrist contractions of various durations (5–30 sec) and intensities (10–50% MVC) (Brasil‐Neto et al. 1993; Balbi et al. 2002). Thus, the magnitude of postexercise depression or lack thereof of spinal excitability depends on the muscle type, contraction intensity and duration, and the state in which it is measured.

Postexercise depression of CMEP amplitude was depressed to a greater extent (*P *<* *0.05, data not shown) immediately postcontraction protocol when recorded at rest (~58%) compared to 5% MVC (~46%). If postexercise depression of CMEPs is partially due to a reduced neurotransmitter release from the corticospinal tract onto the spinal motoneurone (i.e., synaptic fatigue), the descending and/or ascending (afferent) activity may be sufficient to facilitate the release of additional neurotransmitter and thus increase the motoneuronal response. This effect would not be present when CMEPs were assessed at rest (Petersen et al. 2003).

### Postactivation potentiation

PT force increased immediately postcontraction protocol and remained above baseline values for at least 90 sec in *experiments A and B* during both rest and 5% MVC. Other studies showed postactivation potentiation of the elbow extensors (Smith et al. 2011), plantar flexors (Fukutani et al. 2014), thumb abductors (Fukutani et al. 2014) and knee extensors (Dolmage and Cafarelli 1991; Place et al. 2005; Morana and Perrey 2009) following submaximal voluntary contractions at intensities between 20 and 50% MVC. Since the *M*_max_, which is a measure of neuromuscular transmission (i.e., sarcolemma excitability), was not altered the mechanisms for postactivation potentiation likely include a combination of changes in: (1) calcium kinetics (Ismailov et al. 2004), (2) myosin phosphorylation (Grange et al. 1993; Sweeney et al. 1993) and (3) muscle stiffness (Hodgson et al. 2005; MacIntosh 2010).

### Corticospinal excitability and postactivation potentiation relationship

Following the nonfatiguing contraction protocol, as postactivation potentiation increased (1) overall corticospinal excitability increased at rest, but not during a 5% MVC and (2) spinal excitability decreased at rest and during a 5% MVC. Whether these relationships between corticospinal excitability and postactivation potentiation are of functional significance is currently unknown. The relationship between CMEP and postactivation potentiation may have functional consequence. Postactivation potentiation may act to offset postexercise depression of spinal excitability. This relationship may exist due to a reduction in motoneurone output. Motor unit discharge rates have been shown to decrease as postactivation potentiation increases in the tibialis anterior and triceps brachii (Klein et al. 2001; Inglis et al. 2011). Following a contraction protocol, motor unit discharge rates during a 50% MVC of the tibialis anterior were depressed by ~10% whereas postactivation potentiation increased ~120% (Inglis et al. 2011). A similar negative relationship between motor unit discharge rates in the triceps brachii and postactivation potentiation of the elbow extensors has also been shown, albeit at lower percentages of MVC (10–30%) (Klein et al. 2001). The decrease in motor unit discharge rates in these studies was obtained in the absence of fatigue. A reduction in motoneurone output would be consistent with muscle wisdom, enabling the neuromuscular system to potentially maintain a given force with decreased spinal excitability (Bigland‐Ritchie et al. 1983) independent of changes in supraspinal excitability. Furthermore, a reduction in spinal excitability due to postactivation potentiation may help offset or prevent central fatigue (Taylor and Gandevia 2008) by reducing neuromuscular impairment within the muscle (Sale 2004). This pathway may operate to minimize central aspects of neuromuscular fatigue during work or exercise. It should be noted that postactivation potentiation may have no link to the reduction in CMEPs and motor unit discharge rates (i.e., motoneurone output) during and following muscle contraction. An altered motoneurone recruitment behavior during and following muscle contraction itself may lead to reduced motoneurone output.

### Methodological considerations

Because MEPs and CMEPs were not recorded in the same session we could not make a ratio of MEP/CMEP as we (Aboodarda et al. 2015; Pearcey et al. 2016) and others (Gandevia et al. 1999; Gruber et al. 2009) have previously done to demonstrate over all change in supraspinal excitability. However, in the current study the pattern of MEP and CMEP responses and magnitude in change recorded at rest immediately postcontraction protocol were very similar to those recently reported from our lab (Aboodarda et al. 2015). We did not statistically compare MEP or CMEP amplitudes between rest and 5% MVC because corticospinal excitability to a resting and contracting muscle is different due to different portions of the motoneurone pool being recruited. During a contraction, MEP and CMEP responses are much larger compared to those recorded at rest because a portion of the motoneurone pool is active during contraction but quiescent during rest. Subsequently, we inferred about how corticospinal and spinal excitability were state‐dependent based on the changes in the patterns of MEP and CMEP responses and how these changes related to postactivation potentiation. Finally, the pattern of change in MEP and CMEP amplitude may be different immediate following a submaximal contraction protocol if they were measured at higher contraction intensities (Pearcey et al. 2014; Philpott et al. 2015) due to changes in the active portion of the cortex and motoneurone pool.

## Conclusion

The interaction between and changes in corticospinal excitability, spinal excitability and postactivation potentiation following submaximal contractions depended on the state in which they were measured. Postexercise depression of spinal excitability and evoked twitch force postactivation potentiation have longer lasting changes following submaximal contractions than postexercise facilitation of corticospinal excitability. Postactivation potentiation of the muscle may allow for postexercise depression of spinal excitability.

## Conflict of Interest

None declared.
